# Autonomic modulation impacts conduction velocity dynamics and wavefront propagation in the left atrium

**DOI:** 10.1093/europace/euae219

**Published:** 2024-09-04

**Authors:** Shohreh Honarbakhsh, Caroline Roney, Caterina Vidal Horrach, Pier D Lambiase, Ross J Hunter

**Affiliations:** Queen Mary University of London, London, UK; Electrophysiology Department, Barts Heart Centre, Barts Health NHS Trust, W Smithfield, London EC1A 7BE, UK; Queen Mary University of London, London, UK; Queen Mary University of London, London, UK; Electrophysiology Department, Barts Heart Centre, Barts Health NHS Trust, W Smithfield, London EC1A 7BE, UK; Electrophysiology Department, Barts Heart Centre, Barts Health NHS Trust, W Smithfield, London EC1A 7BE, UK

**Keywords:** Conduction velocity, Pivot points, Functional remodelling, Scar, Atrial fibrillation, Autonomic modulation, Ganglionated plexi

## Abstract

**Aims:**

Atrial fibrosis and autonomic remodelling are proposed pathophysiological mechanisms in atrial fibrillation (AF). Their impact on conduction velocity (CV) dynamics and wavefront propagation was evaluated.

**Methods and results:**

Local activation times (LATs), voltage, and geometry data were obtained from patients undergoing ablation for persistent AF. LATs were obtained at three pacing intervals (PIs) in sinus rhythm (SR). LATs were used to determine CV dynamics and their relationship to local voltage amplitude. The impact of autonomic modulation- pharmacologically and with ganglionated plexi (GP) stimulation, on CV dynamics, wavefront propagation, and pivot points (change in wavefront propagation of ≥90°) was determined in SR. Fifty-four patients were included. Voltage impacted CV dynamics whereby at non-low voltage zones (LVZs) (≥0.5 mV) the CV restitution curves are steeper [0.03 ± 0.03 m/s ΔCV PI 600–400 ms (PI_1_), 0.54 ± 0.09 m/s ΔCV PI 400–250 ms (PI_2_)], broader at LVZ (0.2–0.49 mV) (0.17 ± 0.09 m/s ΔCV PI_1_, 0.25 ± 0.11 m/s ΔCV PI_2_), and flat at very LVZ (<0.2 mV) (0.03 ± 0.01 m/s ΔCV PI_1_, 0.04 ± 0.02 m/s ΔCV PI_2_). Atropine did not change CV dynamics, while isoprenaline and GP stimulation resulted in greater CV slowing with rate. Isoprenaline (2.7 ± 1.1 increase/patient) and GP stimulation (2.8 ± 1.3 increase/patient) promoted CV heterogeneity, i.e. rate-dependent CV (RDCV) slowing sites. Most pivot points co-located to RDCV slowing sites (80.2%). Isoprenaline (1.3 ± 1.1 pivot increase/patient) and GP stimulation (1.5 ± 1.1 increase/patient) also enhanced the number of pivot points identified.

**Conclusion:**

Atrial CV dynamics is affected by fibrosis burden and influenced by autonomic modulation which enhances CV heterogeneity and distribution of pivot points. This study provides further insight into the impact of autonomic remodelling in AF.

## Graphical Abstract

**Graphical abstract:**
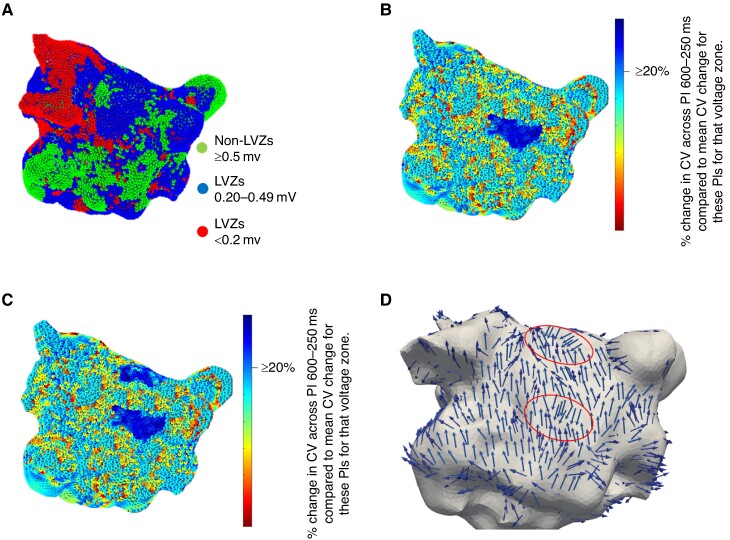

**What’s new?**

- This is the first study to evaluate the impact of autonomic modulation on conduction velocity (CV) dynamics and wavefront propagation in the human left atrium (LA) during atrial pacing in patients undergoing persistent atrial fibrillation (AF) ablation.
- CV dynamics alter depending on the level of scar remodelling and rate-dependent CV (RDCV) slowing sites are predominantly mapped to low voltage zone (0.2–0.49 mV). RDCV slowing sites also co-locate to sites of altered wavefront propagation in sinus rhythm (SR), i.e. pivot points that are potential precursors to re-entry formation. This indicates that the degree of substrate remodelling has a different impact on electrophysiological properties that predispose to re-entry.
- Autonomic modulation pharmacologically and with ganglionated plexi (GP) stimulation impacts CV dynamics in the LA and results in an increase in RDCV slowing sites.
- Autonomic modulation also impacts wavefront propagation in SR and enhances the number of pivot points identified.
- This study provides further insight into how autonomic remodelling in the form of GP contributes to the pathophysiology of AF.

## Introduction

Autonomic remodelling has been proposed to play a pathophysiological role in atrial fibrillation (AF). Animal AF models have a significant increase in sympathetic and parasympathetic nerve density^1,2^ and increased sympathetic nuclei in atrial intrinsic cardiac ganglia.^3^ Temporary suppression of ganglionated plexi (GP) with botulinum toxin in animal models prevents the autonomic remodelling secondary to AF and reduces AF burden.^1^ GP stimulation has also been shown to trigger AF in humans.^4^ In AF patients, there is abnormally increased GP activity, with an increase in the vagal response during GP stimulation.^5^ These findings support the hypothesis that autonomic remodelling could play a potential mechanistic role in AF. However, the mechanisms through which autonomic modulation predisposes to AF in humans remain unclear. Gaining this understanding can potentially aid in formulating ablation strategies for AF ablation.

To establish re-entry, sites of slow conduction are required.^6^ Sites of enhanced conduction velocity (CV) heterogeneity, i.e. rate-dependent CV (RDCV) slowing, have been shown to co-locate to rotational activity in AF and re-entry activity in atrial tachycardia (AT).^7,8^ In animal models and *in vitro* studies, autonomic modulation has been shown to impact CV.^9–11^ The aim of this study was to evaluate the impact of autonomic modulation (both pharmacologically and through GP stimulation) on CV dynamics and wavefront propagation in humans.

## Methods

Patients undergoing catheter ablation for persistent AF (<24 months and no previous AF ablation) were prospectively included. Exclusion criteria were as follows: age <18 years or reversible cause of AF. Patients provided informed consent for their study involvement which was approved by the UK National Research Ethics Service (22/PR/0961). The study was prospectively registered on Clinicaltrial.gov (NCT05633303). Procedures were performed under either conscious sedation or general anaesthetic as per the clinician's and patient’s preference. All patients had anti-arrhythmic drugs and beta-blocker therapy stopped 5 days before the procedure.

### Electrophysiological mapping

#### Scar assessment

Ensite X (Abbott, Chicago, IL, USA) was used as the 3D mapping system. Left atrium (LA) anatomical maps, voltage, and local activation time (LAT) maps were created using the HD-grid mapping catheter (Abbott, Chicago, IL, USA). A decapolar catheter (Boston Scientific, MA, USA) was positioned in the coronary sinus (CS). The Tactiflex ablation catheter (Abbott) was used for ablation.

All patients had a high-density omnipolar voltage map created in AF (see Supplementary material online, Methods). Three voltage zones were defined, very low voltage zones (vLVZs) (<0.2 mV), LVZs (0.2–0.49 mV), and non-LVZs (nLVZs), i.e. normal voltage zones ≥0.5 mV.^7^ Patients then underwent high-frequency stimulation (HFS) to map atrioventricular delay (AVD)-GP sites in AF as described below. Following this, patients were cardioverted to sinus rhythm (SR). All patients had a repeat bipolar voltage (BV) map created in SR with atrial pacing at pacing interval (PI) of 600 ms.

#### CV and CV dynamic assessment

All patients had LAT maps created with fixed atrial pacing at pacing PIs of 600, 400, and 250 ms in SR. LAT maps were created both with endocardial distal CS pacing and left atrial appendage pacing to allow different wavefront directionalities to consider the impact of anisotropy and fibre orientation on CV measurements.

#### Autonomic modulation

All patients underwent autonomic modulation either pharmacologically with isoprenaline and atropine or with GP stimulation. The same patient did not undergo autonomic modulation with both modalities to avoid the interaction of these impacting the results.

##### Pharmacological autonomic modulation

Isoprenaline infusion during SR was utilized to aim for a heart rate increase of 30%. Once this was achieved, LAT maps were then created for each of the three PIs, pacing the distal endocardial CS. Isoprenaline was then stopped and following a waiting period of 20 min ensuring the effect of isoprenaline had washed out, 1000 μg of atropine was given. The LAT maps were then repeated using the same protocol.

##### Autonomic modulation with GP stimulation

GP sites resulting in AVD in AF (AVD-GPs) and triggered ectopy in SR (ET-GPs) were identified. In AF, GP stimulation was performed with HFS at 50 Hz with an output of 100 V through the distal poles of the ablation catheter using a grass S88 stimulator (Astro-Med, West Warwick, RI).^12,13^ Continuous GP stimulation was performed for up to 10 s or until asystole was achieved. Sites that resulted in AVD were tagged as AVD-GP sites on the 3D map. AVD was defined as sites where (i) the RR interval extended by 50% during HFS compared to baseline or (ii) asystole occurred. The baseline RR interval was defined as the mean of 10 RR intervals immediately preceding HFS. The RR interval during HFS was defined as the mean RR interval measured across RR intervals from the first R during HFS and the first R following HFS cessation.^12^

In SR, GP stimulation was performed with synchronized HFS which was again delivered through the distal pole of the ablation catheter (50 Hz, 100 V, 80 ms train duration). HFS was coupled to each paced atrial stimulus to ensure that HFS was delivered within the local atrial refractory period.^13^ Up to 15 trains of HFS were delivered if no ectopy or AF was induced. If ectopy or AF was induced, HFS and pacing were stopped immediately. To ensure ectopy was secondary to HFS and not spontaneously or catheter induced, reproducibility was assessed three times at each site. If AF occurred and this did not spontaneously terminate the patient underwent DCCV to achieve SR. Sites were defined as ET-GP sites if (i) reproducible atrial ectopy and/or (ii) AF was triggered with GP stimulation. These sites were also tagged on the 3D map as ET-GP sites. A minimum of 50 sites were stimulated in the LA body in AF and SR, intentionally avoiding the pulmonary vein (PV) ostium and ensuring adequate LA body coverage.

The tagged ET-GP and AVD-GP sites were then stimulated separately using the above protocol to determine the impact each GP type has on CV dynamics. Due to the artefact generated from HFS stimulation, LAT recordings were not performed during GP stimulation. Thus, LAT recordings were obtained at PI of 600 ms immediately post-GP stimulation. The recordings were limited to 8 s to ensure the effect of the GP stimulation had not ceased.^14,15^ A minimum of 20 recordings were created. This was repeated for the other two PIs (400 and 250 ms). The recordings obtained for each PI were then combined onto the same LAT map on Ensite X. The LAT maps were then utilized to create CV maps for each PI. The maps created pre- and post-autonomic modulation were analyzed to establish the impact on CV dynamics and the distribution of sites of enhanced CV heterogeneity. Co-registered points on the CV maps created pre- and post-autonomic modulation were compared. A point was deemed to co-register if the points were within a geodesic distance of ≤3 mm.

A six-segment anatomical model (roof, posterior, inferior, anterior, lateral, and septal) was also utilized to compare the anatomical distribution of AVD-GP and ET-GP sites.

### Ablation approach

Following mapping, all patients underwent pulmonary vein isolation (PVI) with bilateral wide-area circumferential ablation using radiofrequency ablation (see Supplementary material online, Methods).

### CV methodology

CV was defined as the distance travelled by a wavefront in a unit of time. CV was calculated from electroanatomic mapping data consisting of an LA anatomical mesh and LATs at recording locations projected to the atrial surface. The export data obtained from the 3D mapping system consisted of LA anatomical mesh data and LATs and *xyz* coordinates for each point. LATs were obtained using bipolar electrograms. LATs were all reviewed manually to ensure accuracy in timing (see Supplementary material online, *Figure S1A–D*). The LA anatomical mesh was created utilizing *xyz* coordinates for the vertices and faces that made up the geometry created in Ensite X. The export data were not processed utilizing proprietary 3D mapping system algorithms. These data were processed through an automated proprietary algorithm executed in Matlab (Mathworks, MA, USA) (see Supplementary material online, Methods).

CV measurements and maps were obtained for all three PIs. Differences in the CV measurements at each anatomical location with rate were elicited from these PIs. The voltage at each CV measurement site was also determined. This was used to determine the average CV and CV dynamics in accordance with voltage. To evaluate the presence of heterogeneity in CV dynamics within LVZs and non-LVZs, zones of rate-dependent CV slowing were identified. These were defined as zones exhibiting a reduction in CV between PI = 600 ms and PI = 250 ms of ≥20% of the mean CV reduction seen between these PIs for that voltage zone.

The CV measurements obtained at each pacing site were compared to ensure anisotropy did not impact CV measurements. Sites demonstrating differences in CV measurements due to atrial pacing sites were excluded from the analysis to ensure the CV measurements obtained were not impacted by anisotropy.

### Pivot points

A novel wavefront tracking algorithm developed and executed in Matlab (Mathworks, MA, USA) was used offline to track wavefront propagation (see Supplementary material online, Methods).^16^ Unipolar recordings using the HD-grid catheter were collected by referencing Wilson Central Terminal. A minimum of 30, 30-second unipolar recordings were collected to ensure adequate LA coverage.

The SR wavefront propagation maps were reviewed to identify the presence of pivot points. Pivot points were defined as sites that demonstrated a change in wavefront propagation of ≥90°. The wavefront propagation maps created at PI of 600 ms were reviewed pre- and post-autonomic modulation to determine the impact autonomic modulation had on wavefront propagation and the distribution of pivot points.

### Statistical analyses

Statistical analyses were performed using SPSS (IBM SPSS Statistics, Version 25; IBM Corp, NY, USA). Continuous variables are displayed as mean ± standard deviation (SD) or median (range). Categorical variables are presented as numbers and percentages. The Student *T*-test or Mann–Whitney *U* test was used for the comparison of continuous variables. Fisher’s exact test was used for the comparison of categorical variables. Spearman rank correlation coefficient was determined to assess the relationship between the proportion of LA area occupied by LVZs (0.2–49 mV) and the number of RDCV slowing sites. A *P*-value of <0.05 was deemed significant.

## Results

### Baseline characteristics

**Figure 1:**
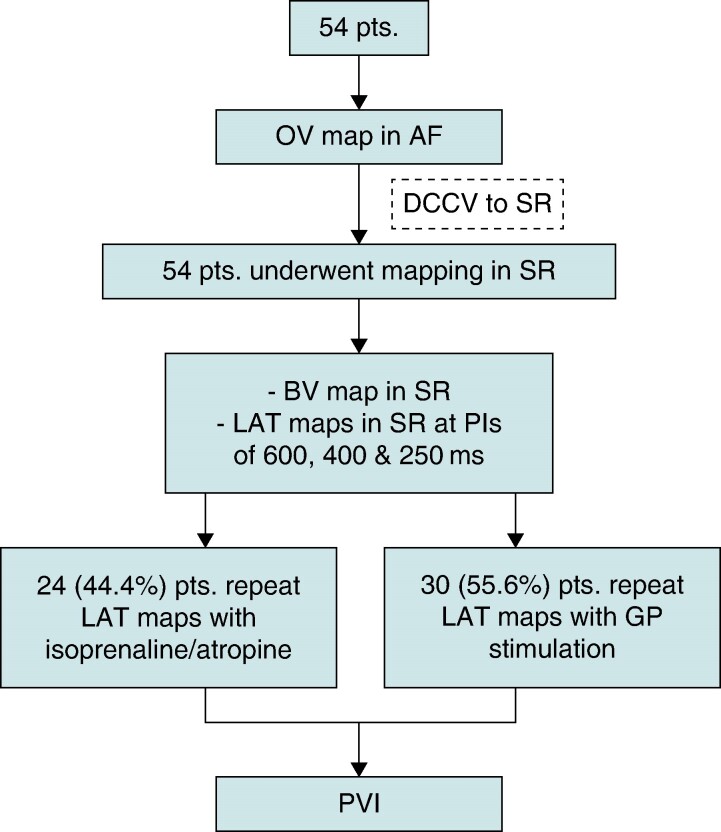
CONSORT flow diagram.

A total of 54 patients were included. Baseline characteristics are demonstrated in *Table 1*. A majority of the procedures were performed under local anaesthetic and sedation (*n* = 30, 55.6%). The average procedure duration was 181.1 ± 35.9 min, with an average fluoroscopy and DAP of 3.1 ± 1.2 min and 44.7 ± 30.8 cGycm2, respectively. No complications were encountered in this cohort. *Figure 1* shows the study flow diagram.

**Table 1:** Baseline characteristics

Baseline characteristics	Cohort *n* = 54
Age (years), mean ± SD	60.6 ± 11.2
Male, *n* (%)	40 (74.1)
Diabetes mellitus, *n* (%)	6 (11.1)
Hypertension, *n* (%)	21 (38.9)
TIA/CVA, *n* (%)	7 (13.0)
Ischaemic heart disease, *n* (%)	8 (14.8)
Cardiac surgery, *n* (%)	1 (1.9)
Cardiomyopathy, *n* (%)	26 (48.1)
BMI (kg/m^2^), *n* (%)	
20–30	33 (61.1)
31–40	18 (33.3)
>40	3 (5.6)
Obstructive sleep apnea, *n* (%)	9 (16.7)
Left ventricular EF ≥ 55%, *n* (%)	25 (46.3)
LA size (mm), *n* (%)	
30–40	17 (31.5)
41–50	29 (53.7)
>50	8 (14.8)
AF duration (months), mean ± SD	19.0 ± 10.3
Previous AT ablation, *n* (%)	4 (7.4)
Cavo-tricuspid isthmus-dependent flutter	4 (100.0)
Current anti-arrhythmic or rate-controlling strategy	
Beta-blockers including Sotalol	44 (81.5)
Amiodarone	22 (40.7)
Flecainide	7 (13.0)
Calcium channel blocker	2 (3.7)
Digoxin	7 (13.0)
Current anticoagulation strategy	
Warfarin	0 (0.0)
Direct oral anticoagulants	54 (100.0)

EF, ejection fraction; TIA/CVA, transient ischaemic attack/cerebrovascular accident.

### CV and CV dynamics and the relationship with voltage

**Figure 2:**
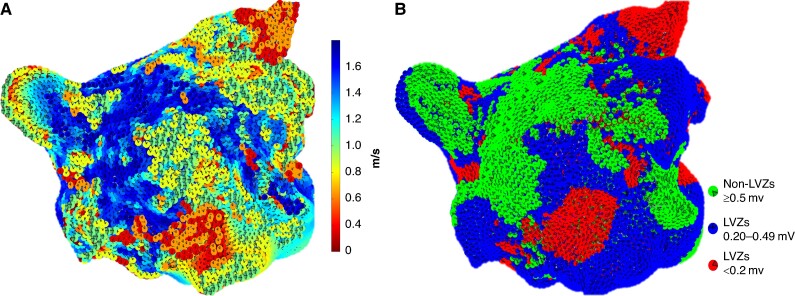
Demonstrates (*A*) a CV map that demonstrates differences in CV depending on the underlying voltage. (*B*) A bipolar voltage map was recreated in Matlab where green dots represent nLVZs (≥0.5 mV), blue dots represent LVZs (0.20–0.49 mV), and red dots represent vLVZs (<0.2 mV).

A total of 1 540 296 LAT and voltage points were analyzed (28 524 ± 4575 points per patient). The average CV obtained at a PI of 600 ms was 1.24 ± 0.25 m/s. The CV obtained across the three voltage zones at this PI was significantly different (*Table 2* and *Figure 2A* and *B*). The mean CV was higher in areas of nLVZs compared to areas of LVZ <0.5 mV (1.57 ± 0.15 m/s vs. 0.97 ± 0.25 m/s, *P* < 0.001) (*Figure 2A* and *B*). LVZs <0.5 mV were predominantly seen on the posterior wall (32.5%), inferior wall (25.2%), and anterior wall (20.1%). CVs of <1 m/s at a PI of 600 ms spatially co-located to the distribution of LVZs <0.5 mV and thereby also predominantly seen on the posterior wall (30.4%), inferior wall (24.3%), and anterior wall (19.5%).

**Table 2:** The CV changes across the three pacing intervals stratified in accordance with the three pre-defined voltage zones with and without autonomic modulation

	Non-LVZs (≥0.50 mV)	LVZs (0.2–0.49 mV)	vLVZs (<0.2 mV)	*P*-value
Without autonomic modulation				
CV at 600 ms, m/s, mean ± SD	1.57 ± 0.15	1.07 ± 0.23	0.57 ± 0.22	<0.001
CV change, m/s, mean ± SD				
600–400 ms PIs	0.03 ± 0.03	0.17 ± 0.0.09	0.03 ± 0.01	<0.001
400–250 ms PIs	0.54 ± 0.09	0.25 ± 0.11	0.04 ± 0.02	<0.001
RDCV slowing sites %	21.5	78.5	0	<0.001
Change in CV at RDCV slowing sites, m/s, mean ± SD				
600–400 ms PIs	0.06 ± 0.03	0.19 ± 0.07		<0.001
400–250 ms PIs	0.64 ± 0.14	0.31 ± 0.08		<0.001
With autonomic modulation				
Atropine				
CV at 600 ms, m/s, mean ± SD	1.58 ± 0.12	1.04 ± 0.21	0.58 ± 0.13	<0.001
CV change, m/s, mean ± SD				
600–400 ms PIs	0.05 ± 0.03	0.18 ± 0.10	0.02 ± 0.02	<0.001
400–250 ms PIs	0.53 ± 0.12	0.25 ± 0.12	0.05 ± 0.03	<0.001
Isoprenaline				
CV at 600 ms, m/s, mean ± SD	1.59 ± 0.14	1.09 ± 0.19	0.56 ± 0.17	<0.001
CV change, m/s, mean ± SD				
600–400 ms PIs	0.18 ± 0.04	0.31 ± 0.10	0.03 ± 0.02	<0.001
400–250 ms PIs	0.60 ± 0.12	0.31 ± 0.13	0.06 ± 0.03	<0.001
GP stimulation				
AVD-GP				
CV at 600 ms, m/s, mean ± SD	1.57 ± 0.16	1.07 ± 0.11	0.55 ± 0.10	<0.001
CV change, m/s, mean ± SD				
600–400 ms PIs	0.19 ± 0.07	0.28 ± 0.07	0.03 ± 0.02	<0.001
400–250 ms PIs	0.60 ± 0.11	0.32 ± 0.11	0.04 ± 0.02	<0.001
ET-GP				
CV at 600 ms, m/s, mean ± SD	1.58 ± 0.13	1.00 ± 0.20	0.56 ± 0.11	<0.001
CV change, m/s, mean ± SD	0.18 ± 0.07	0.26 ± 0.07	0.03 ± 0.02	<0.001
600–400 ms PIs				
400–250 ms PIs	0.60 ± 0.13	0.32 ± 0.12	0.05 ± 0.02	<0.001

AVD, atrioventricular delay; CV, conduction velocity; ET, ectopy triggered; GP, ganglionated plexi; LVZs, low voltage zones; PIs, pacing intervals; RDCV, rate-dependent conduction velocity.

**Figure 3:**
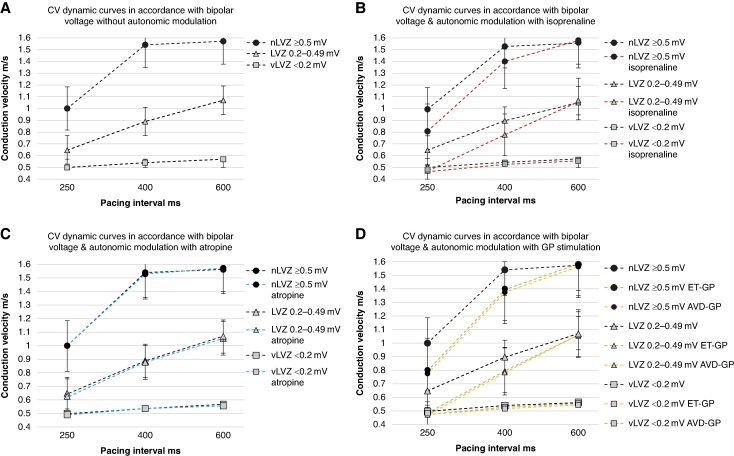
Demonstrate CV dynamics curves with the change in CV over pacing intervals of 600, 400, and 250 ms in accordance with voltage (*A*) without autonomic modulation (dashed black line), (*B*) with autonomic modulation with isoprenaline (dashed red line), (*C*) with autonomic modulation with atropine (dashed blue line), and (*D*) with autonomic modulation with GP stimulation (ET-GP—dashed green line and AVD-GP sites—dashed orange line).

The CV dynamic curves obtained at the three PIs were different across the three pre-defined voltage zones (*Figure 3A–D* and *Table 2*). vLVZs (<0.2 mV) demonstrated flat curves with minimal change in CV. In contrast, LVZs (0.2–0.49 mV) showed a steady change in CV with rate. nLVZs (≥0.5 mV) demonstrated a minimal change in CV between 600 and 400 ms, while the CV change between 400 and 250 ms was greater.

RDCV slowing sites were identified in all patients, with an average of 2.8 ± 1.1 RDCV slowing sites per patient with a total of 149 RDCV slowing sites identified. Correlating the RDCV sites with the underlying voltage, RDCV sites were predominantly seen in LVZs (0.2–0.49 mV) (117/149, 78.5%). No RDCV sites were demonstrated in vLVZs (<0.2) and the remaining 32 (21.5%) RDCV slowing sites were seen in nLVZs (≥0.5 mV). There was a positive correlation between the proportion of the LA area occupied by LVZs (0.2–0.49 mV) and the number of RDCV slowing sites identified (*r_s_* = 0.91). CV dynamic curves for the RDCV slowing sites showed different patterns for LVZs (0.2–0.49 mV) and nLVZs (≥0.5 mV), whereby for LVZs (0.2–0.49 mV), there was a steady decrease in CV over all three PIs resulting in broader curves. The RDCV slowing sites in nLVZs showed the greatest decrease in CV between PIs of 400 and 250 ms with minimal change in CV between PIs 600 and 400 ms, resulting in a steeper curve (*Table 2*).

### Impact of autonomic modulation on CV and CV dynamics

#### Pharmacological autonomic modulation

Out of the 54 patients, 24 (44.4%) patients had CV maps created with autonomic modulation with isoprenaline and atropine. While isoprenaline impacted the CV dynamic curves through enhancing the rate dependency slowing, atropine did not (*Figure 3A–D* and Tables *2* and *3*). In nLVZs (≥0.5 mV), isoprenaline caused a significant increase in the change in CV across all PIs when compared to CV change without isoprenaline (0.15 ± 0.06 m/s increased CV slowing PI 600–400 ms; *P* < 0.001 and 0.06 ± 0.10 m/s increased CV slowing PI 400–250 ms; *P* = 0.02). This was also seen in LVZs (0.2–0.49 mV) with a 0.14 ± 0.13 m/s increase in CV slowing between PI 600 and 400 ms; *P* < 0.001 and 0.06 ± 0.10 m/s between PI 400 and 250 ms; *P* < 0.001). Isoprenaline did not impact CV dynamic curves in vLVZs (<0.2 mV). The change in CV dynamic curves with isoprenaline also resulted in an enhancement in the proportion of RDCV slowing sites identified (2.8 ± 1.1 pre-isoprenaline vs. 5.5 ± 1.0 post-isoprenaline; *P* < 0.001). The proportion of RDCV slowing sites did not change with atropine (2.8 ± 1.1 pre-atropine vs. 2.9 ± 1.0 post-atropine; *P* = 0.43).

**Table 3:** The change in CV over the three PIs with and without autonomic modulation pharmacologically and GP stimulation

	No Iso	Iso	*P*-value	No Atro	Atro	*P*-value	No GP stim.	GP stim.	*P*-value
nLVZs (≥0.50 mV)									
CV change, m/s, mean ± SD									
600–400 ms PIs	0.03 ± 0.03	0.18 ± 0.04	<0.001	0.03 ± 0.02	0.05 ± 0.03	0.78	0.03 ± 0.02	0.19 ± 0.07	<0.001
400–250 ms PIs	0.54 ± 0.09	0.60 ± 0.12	0.02	0.54 ± 0.09	0.53 ± 0.12	0.83	0.54 ± 0.09	0.60 ± 0.11	<0.001
LVZs									
(0.2–0.49 mV)									
CV change, m/s, mean ± SD									
600–400 ms PIs	0.17 ± 0.12	0.31 ± 0.10	<0.001	0.17 ± 0.12	0.18 ± 0.10	0.63	0.17 ± 0.12	0.28 ± 0.07	<0.001
400–250 ms PIs	0.25 ± 0.11	0.31 ± 0.13	<0.001	0.25 ± 0.11	0.25 ± 0.12	0.82	0.25 ± 0.11	0.32 ± 0.11	<0.001
vLVZs <0.2 mV									
CV change, m/s, mean ± SD									
600–400 ms PIs	0.03 ± 0.01	0.03 ± 0.02	0.54	0.03 ± 0.01	0.02 ± 0.02	0.42	0.03 ± 0.02	0.03 ± 0.02	0.43
400–250 ms PIs	0.04 ± 0.02	0.06 ± 0.03	0.52	0.04 ± 0.02	0.05 ± 0.03	0.31	0.06 ± 0.03	0.05 ± 0.02	0.38

Atro, atropine; GP stim., ganglionated plexi stimulation; Iso, isoprenaline; nLVZ, non-low voltage zone; PIs, pacing intervals.

Pharmacological autonomic modulation with either isoprenaline (difference between co-registered BV points 0.08 ± 0.03 mV; *P* = 0.56) or atropine (difference between co-registered BV points 0.08 ± 0.02 mV; *P* = 0.68) did not impact the underlying BV when compared to co-registered points on maps pre-pharmacological autonomic modulation.

#### Autonomic modulation with GP stimulation

**Figure 4:**
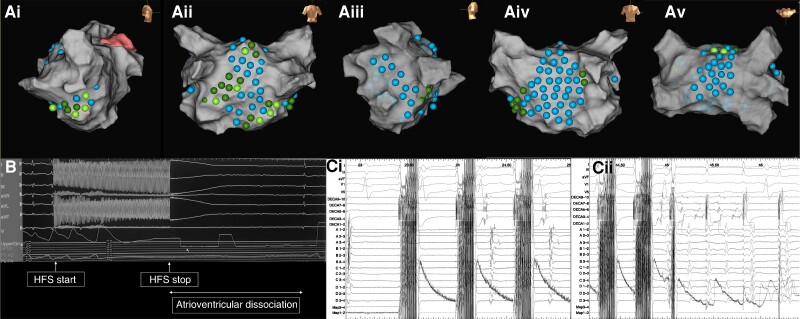
Demonstrates an LA geometry from one patient in (*Ai*) right lateral view, (*Aii*) posterior–anterior (PA) view, (*Aiii*) left lateral view, (*Aiv*) anterior–posterior (AP) view, and (*Av*) roof view that highlights sites where HFS was performed. Blue lesions highlight sites where HFS did not elicit a response that met the criteria for an ET-GP site or an AVD-GP site. Sites where HFS was performed and resulted in a response that indicated an AVD-GP site is highlighted with a dark green lesion. Sites where HFS was performed and resulted in a response that indicated an ET-GP site is highlighted with a light green lesion. (*B*) Atrioventricular dissociation with HFS stimulation in AF at an AVD-GP site. (*Ci* and *Cii*) Reproducible atrial ectopy with HFS stimulation in SR at an ET-GP site.

**Figure 5:**
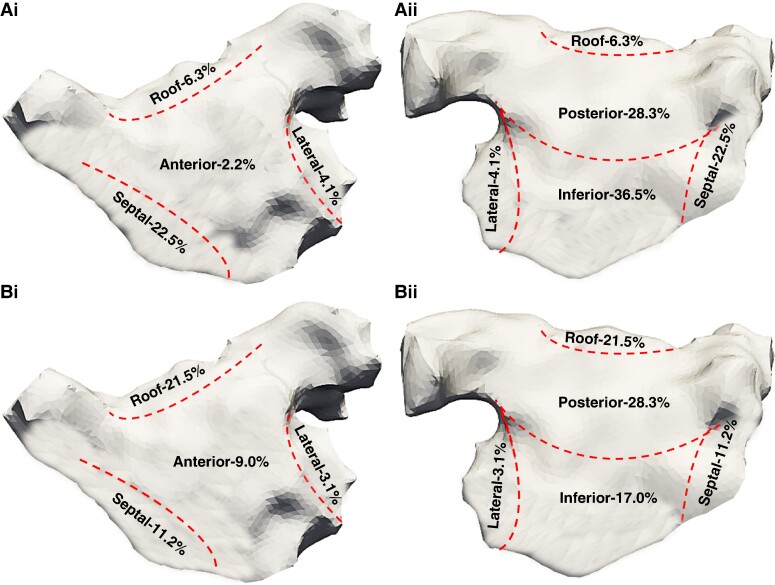
The anatomical distributions of GP sites across a six-segment model (roof, anterior, septum, lateral, posterior, and inferior) in the distribution of AVD-GP sites in (*Ai*) AP and (*Aii*) PA view and distribution of ET-GP sites in (*Bi*) AP and (*Bii*) PA view.

Out of the 54 patients, 30 (55.6%) patients had autonomic modulation performed with GP stimulation. A total of 3202 sites underwent HFS in AF and SR with an average of 106.7 ± 5.2 sites per patient and 53.4 ± 3.2 sites per map. All patients had ET-GP and AVD-GP sites mapped (*Figure 4A–C*). Out of the 1663 sites stimulated in AF, 341 (20.5%) sites demonstrated an AVD response with an average of 11.4 ± 4.1 AVD-GP sites per patient. AVD-GP sites were predominantly mapped to inferior (*n* = 125, 36.7%), posterior (*n* = 97, 28.4%), and septal wall particularly under the right lower PV (*n* = 77, 22.6%) (*Figure 5A* and *B*). Out of the 1539 sites stimulated in SR, 223 (14.5%) sites demonstrated an ET response with an average of 7.4 ± 5.7 ET-GP sites per patient. ET-GP sites were predominantly mapped to the posterior wall, particularly near the left lower PV (*n* = 63, 28.3%), inferior (*n* = 38, 17.0%), and roof (*n* = 52, 23.3%) (*Figure 5A* and *B*). AVD-GP sites occupied a larger area of the anatomical sites compared to ET-GP sites.

**Figure 6:**
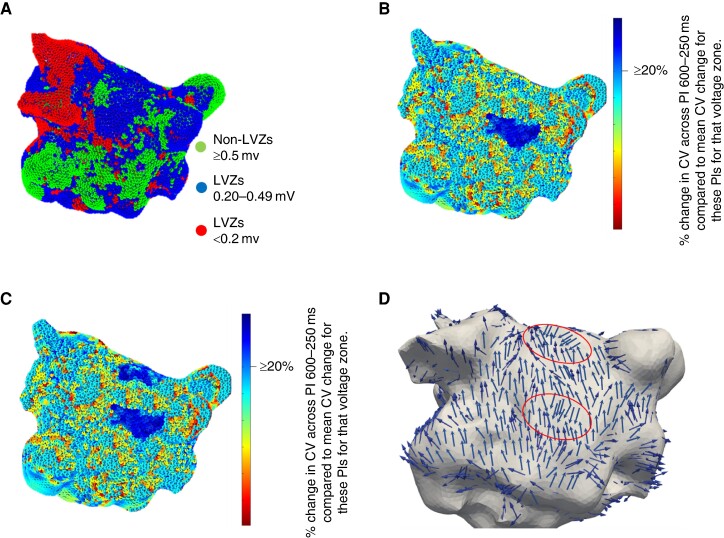
(*A*) a bipolar voltage map in a posterior–anterior (AP) view recreated in Matlab where green dots represent nLVZs (≥0.5 mV), blue dots represent LVZs (0.20–0.49 mV), and red dots represent vLVZs (<0.2 mV). (*B*) CV map without autonomic modulation. The CV map is created by taking the reduction in CV between 600 and 250 ms at a site and then determining the percentage reduction when compared to the average reduction in CV between 600 and 250 ms for that voltage zone. Blue highlights sites with a CV change of ≥20%, i.e. RDCV slowing sites. An RDCV slowing site was identified on the mid-posterior wall which co-locates to an LVZ (0.2–0.49 mV). (*C*) CV map following autonomic modulation with GP stimulation created using the above methodology. The RDCV slowing site identified on the mid-posterior wall without autonomic modulation is present. There is an additional RDCV slowing site at the mid-roof following autonomic modulation with GP stimulation. (*D*) Wavefront propagation map created post-GP stimulation where the arrows highlight the wavefront propagation along the posterior wall. The arrows indicate two sites where there is a change in wavefront propagation of >90° that highlight pivot points (area encircled by a red circle). The pivot point co-locates to a site of LVZs (0.2–0.49 mV) and the RDCV slowing sites. The pivot point on the mid-posterior wall was present pre-GP stimulation. The additional pivot point on the mid-roof was present post-GP stimulation.

The impact on CV dynamics was consistent between ET-GP and AVD-GP sites. GP stimulation resulted in an enhancement in rate dependency slowing across the three PIs (*Figure 3A–D* and *Tables 2* and *3*). The enhancement in rate dependency slowing seen was consistent with that seen with isoprenaline. Autonomic modulation with GP stimulation including both ET-GP and AVD-GP sites resulted in a significant increase in CV change across all PIs in nLVZs (≥0.5 mV) (0.14 ± 0.09 m/s increased CV slowing PI 600–400 ms; *P* < 0.001 and 0.06 ± 0.04 m/s increased CV slowing PI 400–250 ms; *P* = 0.01). This was also seen in LVZs (0.2–0.49 mV) with a 0.11 ± 0.10-m/s increase in CV slowing between PI 600 and 400 ms (*P* < 0.001) and 0.07 ± 0.09 m/s between PI 400 and 250 ms (*P* = 0.001). GP stimulation did not impact CV dynamic curves in vLVZs (<0.2 mV). The change in CV dynamic curves with GP stimulation resulted in an enhancement in the proportion of RDCV slowing sites identified (2.8 ± 1.1 pre-GP stimulation vs. 5.6 ± 1.3 post-GP stimulation; *P* < 0.001) (representative *Figure 6A–D*).

Autonomic modulation with GP stimulation (difference between co-registered BV points 0.08 ± 0.04 mV; *P* = 0.64) did not impact the underlying BV when compared to co-registered points on maps pre-autonomic modulation with GP stimulation.

The autonomic modulation impact on CV dynamics was consistent across all patients regardless of their underlying co-morbidities.

### Wavefront tracking

In the 54 patients, a total of 172 pivot points (3.2 ± 1.1 pivot points per patient) were identified on the wavefront propagation maps created in SR. Out of these pivot points, 146 (84.9%) co-located to sites of LVZ (0.2–0.49 mV) and 138 (80.2%) co-located to sites of RDCV slowing. Pivot points that were mapped to RDCV slowing sites were all mapped to LVZs (0.2–0.49 mV). When reviewing the 24 wavefront propagation maps created with autonomic modulation with isoprenaline, an additional 31 pivot points (3.3 ± 1.0 pre-isoprenaline vs. 4.6 ± 1.2 post-isoprenaline; *P* = 0.001) were identified that were not seen on the wavefront propagation maps without autonomic modulation with isoprenaline (representative *Figure 6A–D*). GP stimulation resulted in an additional 36 pivot points (3.3 ± 1.0 pre-GP stimulation vs. 4.8 ± 1.1 post-GP stimulation; *P* < 0.001). These additional pivot points were mapped to RDCV slowing sites and to LVZ (0.2–0.49 mV).

## Discussion

This is the first study to evaluate the impact of autonomic modulation on CV dynamics and wavefront propagation in the human LA during atrial pacing in patients undergoing persistent AF ablation. In this study, we have demonstrated that autonomic modulation pharmacologically and with GP stimulation results in enhanced CV slowing and an increase in sites of CV heterogeneity, i.e. RDCV slowing sites. Furthermore, autonomic modulation impacts wavefront propagation and enhances the number of pivot points that are a potential precursor to re-entry formation.^17^

### Atrial scar assessment

In this study, scar assessment in the LA was performed using contact mapping with voltage assessment. This modality was utilized as this is a well-validated methodology and widely used in mechanistic substrate studies^7,8,18^ and to guide substrate modification ablation strategies.^19^ The use of cardiac MRI to evaluate left atrial scar is being widely evaluated.^20–22^ However, the LGE detection method that should be utilized is not well established. A recent study has shown discordance between LVZs, slow conduction areas, and LGE-MRI in the LA irrespective of the LGE detection method.^23^ Therefore, in this study, scar assessment in the LA was performed using voltage assessment with contact mapping.

### The relationship of CV and CV dynamics with voltage

As shown in previous studies, CV is lower in areas of scar compared to healthy tissue^7,8^ emphasizing a link between structural and electrical remodelling. In this study, CV dynamics differ in accordance with the underlying voltage consistent with other studies.^7^ Normal voltage demonstrated steep CV dynamic curves with a minimal change in CV between PI of 600 and 400 ms and a significant change in CV between PI of 400 and 250 ms. In LVZs (0.2–0.49 mV), the CV dynamic curves were broad with a steady change in CV across the three PIs, while for vLVZs (<0.2 mV), the curves were flat with a minimal change in CV with rates. The difference in CV dynamic curves between LVZs (0.2–0.49 mV) and vLVZ (<0.2 mV) highlights that there are differences in scar effects on electrophysiological properties.

The change in CV dynamics with structural remodelling is likely due to fibroblast–myocyte coupling and replacement of myocardial tissue by fibrosis which occurs in the context of structural remodelling, and which has been shown to impact CV and promote CV heterogeneity.^24,25^ It is plausible that the differences in CV dynamics between LVZ (0.2–0.49 mV) and vLVZ (<0.2 mV) are secondary to LVZ (0.2–0.49 mV) tissue being healthy enough that it can adapt to rate while vLVZ (<0.2 mV) has minimal conduction reserve. The differences seen in CV dynamics in these LVZs in response to autonomic modulation could also be due to variation in the type of fibrotic strands,^25^ fibroblast myocyte coupling,^24^ interstitial collagen strands,^26^ and the effect of axial resistance.^27^

The different levels of LVZs also had variable CV heterogeneity, i.e. RDCV slowing sites. vLVZ (<0.2 mV) did not harbour any RDCV slowing sites while they were predominantly mapped to LVZ (0.2–0.49 mV). RDCV slowing sites co-locate to rotational drivers in AF and re-entry activity in AT^7,8^ and in this study co-locate to pivot points. These findings highlight that all scar sites may not be mechanistically important to AF maintenance and could be used to help stratify substrate modification ablation strategy to scar that harbours CV heterogeneity.^28^ A proportion of RDCV slowing sites were identified in nLVZs (≥0.5 mV); however, the CV dynamic curves for these sites were steeper compared to broader for the RDCV slowing sites seen in LVZ (0.2–0.49 mV). Broad CV dynamic curves have an alteration in activation vector and arcing with accelerates rates which may reflect rate-dependent conduction block in certain directions^29^ that can promote re-entry.^30^ This is consistent with the findings in this study, whereby pivot points are only co-located to RDCV slowing sites mapped to LVZs (0.2–0.49 mV). The role of CV heterogeneity in AF is further supported by a recent study which showed that more local directional heterogeneity in CV was seen in SR in AF patients compared to non-AF patients.^31^

### Impact of autonomic modulation on CV and CV dynamics

Autonomic remodelling has been proposed to play a mechanistic role in AF. In animal models, AF results in sympathetic and vagal hyperinnervation.^32,33^ In patients with chronic AF, there is an increase in atrial sympathetic nerve density.^34^ Autonomic modulation through GP ablation or pharmacological autonomic blockade reverses the acute remodelling and eliminates AF inducibility.^35^ The incidence of atrial arrhythmias reduces with downregulation of autonomic nervous system innervation.^36,37^ In human studies, the vagal response seen with GP stimulation is significantly higher in patients with AF compared to patients without AF.^5^ Hence, autonomic GP ablation has been utilized as an ablation strategy for AF.^13,38^

Even though it has been established that autonomic remodelling occurs in the context of AF and is a potential AF therapeutic target, it has not yet been established the impact autonomic modulation has on electrophysiological properties in humans. For the first time, we have established that autonomic modulation pharmacologically and through GP stimulation impacts CV dynamics by enhancing CV slowing with rate and sites of CV heterogeneity. Intra-atrial conduction delay is a feature that would favour re-entry formation.^6,39^ Further to this, autonomic modulation resulted in an enhancement in RDCV slowing sites which have been shown to spatially co-locate to rotational drivers in AF and predictive of rotational drivers resulting in AF termination on ablation.^7^ Autonomic modulation also impacted wavefront propagation resulting in an increased number of pivot points. Thereby, in this study, we have shown that autonomic modulation enhances an environment that predisposes to re-entry formation and provides a mechanism for which autonomics have been shown to promote rotor formation and enhance rotor frequency.^40,41^ These findings further highlight a potential target for ablation in addition to substrate modification. A recent animal study has also shown that sympathetic nervous system modulation with renal denervation does impact CV measurements in the LA further supporting a link between CV and autonomic modulation.^42^

#### Pharmacological autonomic modulation

While isoprenaline had an impact on CV dynamics by increasing the CV slowing at increased heart rates and the number of RDCV slowing sites atropine did not. It is not clear why atropine did not have an impact on CV dynamics, as it is well established that there is vagal hyperinnervation in AF.^32^ It is plausible that the sympathetic remodelling seen in AF influences CV dynamics rather than the parasympathetic remodelling. In animal models, sympathetic stimulation rather than vagal stimulation resulted in an increase in atrial CV^43^ highlighting that sympathetic stimulation plays a more crucial role with regard to CV dynamics as shown in this study. Further to this, autonomic modulation with isoprenaline also impacted wavefront propagation and increased the number of pivot points. This was not seen with atropine. In animal models, sympathetic stimulation results in an increase in AF inducibility.^43^ Isoprenaline has also shown to shorten the action potential duration (APD) and effective refractory period (ERP),^9^ and with the link between APD and CV restitution,^44^ this could be account for the change in CV dynamics seen with isoprenaline. Further to this, *ex vivo* studies have shown that isoprenaline enhances tissue excitability which can impact CV.^10^ This study has shown that these findings are consistent in humans.

#### GP stimulation

Chemical and electrical GP stimulation has been shown to induce AF through PV focal firing.^4,45^ In animal models, GP ablation suppresses or eliminates GP-triggered rapid firings.^46^ These findings have provided the grounds for GP ablation as an additional ablation strategy for AF. GP consists of both parasympathetic and sympathetic components. The effect of PV triggers by GP stimulation has been shown to be blocked by both atropine and atenolol suggesting that this effect is mitigated by both autonomic components.^47^ Studies have also evaluated the effect of parasympathetic and sympathetic nerve innervation on atrial electrophysiological properties. Electrical or chemical parasympathetic and sympathetic stimulation shortens the atrial ERP and the wavelength of the cardiac impulse,^48,49^ which in turn increases the probability of multiple re-entrant circuits existing simultaneously and thereby enhances AF maintenance.^50^

Pharmacological autonomic modulation in this study showed an effect with isoprenaline but not atropine. This therefore potentially suggests that the sympathetic component of GP plays a role in CV dynamics. However, this does not exclude the role of the parasympathetic component on the grounds that blocking the parasympathetic effect on CV dynamics with atropine might not result in an upward shift in CV dynamic curves because of the lack of additional conduction reserve to do so. To fully establish the role of the parasympathetic component, it would require pharmacological evaluation with drugs that rather enhance this effect than blocking it.

Low-intensity GP stimulation prolongs ERP and action potential duration in ventricular tissue.^11^ GP ablation significantly prolongs the atrial ERP.^35^ In *in vitro* studies, HFS results in a heterogenous response with shortening of action potential in some cells but lengthening of the action potential and development of early after depolarization in other cells.^51^ This could account for the differences seen in the response to GP stimulation seen in different voltage zones.

In this study, we aimed to differentiate ET-GP and AVD-GP sites in the LA body and determine whether they had a different impact on CV dynamics. HFS was intentionally restricted to the LA body avoiding the PV ostium to exclude GP sites that are targeted within the WACA lines. The anatomical distribution of ET-GP and AVD-GP sites in the LA body overlapped suggesting that the same GP sites can elicit different responses whether mapped in SR or AF and are not separate entities. Previous studies report differences in the anatomical distribution of ET-GP and AVD-GP sites; however, they also performed HFS around the PV ostium.^13^ In this study, while the anatomical distribution of the ET-GP and AVD-GP sites was consistent, AVD-GP sites did cover a larger area compared to ET-GP sites and a greater proportion of HFS sites demonstrated an AVD-GP response. The smaller proportion of HFS sites eliciting an ET-GP response is most likely due to the exclusion of the stimulation of the PV ostium as previous studies have shown that the ET-GP sites are predominantly mapped to the PV ostium.^13^ These have suggested that AVD-GP sites are less specific to the pathogenesis of AF.^13^ However, in our study, the impact autonomic modulation through stimulation of ET-GP and AVD-GP sites had on CV dynamics was comparable which is not surprising due to the spatial relationship between the GP sites.

## Limitations

The study has evaluated a potential role for RDCV slowing sites in stratifying scar that is mechanistically important in AF. The impact of the use of this modality in guiding substrate modification ablation strategies in AF needs to be further evaluated in randomized controlled trials. This study also determined the impact of autonomic modulation with GP stimulation on CV dynamics but did not evaluate its role with regard to AF mechanisms and as an ablation target. Further studies are required to evaluate the role of autonomic modulation with GP stimulation on AF mechanisms and as an ablation target in persistent AF.

The export data obtained from the 3D mapping system consisted of LA anatomical mesh data and LATs and *xyz* coordinates for each point. These data were processed through a proprietary automated algorithm executed in Matlab to obtain CV measurements. To generate the wavefront propagation maps, raw unipolar electrogram data obtained for each point, the corresponding *xyz* coordinates for the points, and LA anatomical mesh data were processed through a proprietary automated algorithm in Matlab to generate the wavefront propagation maps. The export data can be obtained from a majority of 3D mapping systems. The export data were not processed utilizing proprietary 3D mapping system algorithms, and thereby, the study findings are unlikely to be impacted by the 3D mapping system that was used. However, it would be beneficial to evaluate these results using other 3D mapping systems.

In this study, pharmacological modulation was performed separately with isoprenaline and atropine. The combination of isoprenaline and atropine was not tested; however, with the lack of effect seen with atropine on its own, the combination is unlikely to influence the CV dynamics; however, this requires further evaluation.

Mapping in this study was performed using sequential mapping with an HD-grid catheter. To allow effective tracking of the wavefront using the novel algorithm, the sequential recording overlapped, ensuring each segment had several overlapping recordings in each direction. Even though this approach will allow evaluation of wavefront propagation over segments, it will not allow comparison of wavefront propagation across opposite anatomical surfaces. To achieve this, global simultaneous mapping with whole-chamber basket catheters would be required which are unfortunately now commercially unavailable.

In this study, the impact of autonomic modulation on CV dynamics and wavefront propagation in the LA was evaluated in patients undergoing ablation for persistent AF. Patients with persistent AF and no previous left atrial ablation were included to ensure previous ablation particularly ablation outside of the PVs did not impact the findings of the study. Persistent AF patients were also the focus as the role of GP ablation as an additional ablation target beyond the PVs is being evaluated in these patients. Further evaluation in other AF patient groups would be of benefit to ensure these findings are applicable to all AF types.

## Conclusions

There is an interaction between autonomic modulation in the form of GP stimulation and electrical remodelling providing further insight into the pathophysiology of AF. This study has shown that CV dynamics alter depending on the level of scar remodelling and sites of enhanced CV heterogeneity, i.e. RDCV slowing sites are predominantly mapped to LVZ (0.2–0.49 mV). CV heterogeneity sites co-located to sites of altered wavefront propagation, i.e. pivot points that are potential precursor to re-entry. This indicates that the degree of substrate remodelling has a different impact on electrophysiological properties that predispose to re-entry. These findings can potentially aid in the stratification of scar that is mechanistically important in AF during substrate modification ablation. Autonomic modulation both pharmacologically and through GP stimulation impacts CV dynamics by increasing the CV slowing seen with rate and enhances sites of CV heterogeneity and distribution of pivot points, providing further insight into how autonomic remodelling contributes to the pathophysiology of AF.

## Supplementary Material

euae219_Supplementary_Data

## Data Availability

The data underlying this article will be shared on reasonable request to the corresponding author.
