# Supplementary material for: Autonomic modulation impacts conduction velocity dynamics and wavefront propagation in the left atrium
Source: Europace. 2024 Sep 4;26(9):euae219. doi: 10.1093/europace/euae219 (PMC11372476; doi:10.1093/europace/euae219)
Supplement: euae219_Supplementary_Data [file euae219_supplementary_data.zip › Supplemental Figure Legends.docx]

***Supplemental Figure Legends***

***Figure 1A-B-*** *Demonstrates replicas of the local activation time (LAT) maps created in Ensite X. These maps were created using the local activation times for each point, xyz coordinates for each point and left atirum anatomical mesh data. This data was then processed through an automated proprietary algorithm executed in Matlab to obtain conduction velocity measurements. As per the scale, red highlights sites of later activity whilst blue highlights sites of early activity* ***Ai-iii-*** *LAT maps in an anterior-posterior view created with atrial pacing from left atrial appendage.* ***Bi-iii-*** *LAT maps in a posterior-anterior view created with atrial pacing from endocardial distal CS.*
