# Supplementary material for: Autonomic modulation impacts conduction velocity dynamics and wavefront propagation in the left atrium
Source: Europace. 2024 Sep 4;26(9):euae219. doi: 10.1093/europace/euae219 (PMC11372476; doi:10.1093/europace/euae219)
Supplement: euae219_Supplementary_Data [file euae219_supplementary_data.zip › Supplemental Method.docx]

**Supplemental Methods**

1. ***Scar assessment***

Ensite X (Abbott, Chicago, IL, USA) was used as the 3D mapping system. Left atrial (LA) anatomical maps, voltage and local activation time (LAT) maps were created using the HD-grid mapping catheter Points that were ≥5mm from the geometry surface were filtered as not being in contact with the myocardium, and points acquired were respiratory gated to optimize the accuracy of anatomical localization. A minimum of 5000 voltage points were collected per patient with the aim to ensure adequate atrial coverage. The interpolation threshold was set to 5mm for surface color projection and points were collected aiming for complete LA coverage (i.e., with no area >5mm from a data point).

For BV assessment, the bipole obtained from the electrodes along the spline of the HD-grid was used. The window of interest (WOI) was set to exclude the QRS complex. The number of atrial beats within the WOI was dependent on the AF cycle length and the ventricular rate in AF. The largest peak-to-peak voltage point was then determined from all the atrial beats in the WOI. Following this, all peak-to-peak voltage points identified within a 1mm sphere were then identified. The best duplicate algorithm was then applied whereby the timing annotations of all these points were analyzed. The extreme outliers were excluded. The remaining points were analyzed, and the average timing annotation was calculated. The point with the largest voltage with timing near the average timing was used for the final BV. For OV assessment, signals were obtained from three non-colinear electrodes that make up a clique. These are used to calculate the BV in all directions over 360 degrees. The BV with the largest bipolar peak-to-peak voltage is then used to compute a local virtual bipolar signal that represents the OV. Following this, all peak-to-peak voltage points identified within a 1mm sphere were identified. The voltage point with the highest OT certainty (numeric value ranging from 0-1 indicating how certain the calculated activation direction is) and largest voltage amplitude is then used for the final OV.

1. ***Ablation approach***

Following CV assessment, all patients underwent pulmonary vein isolation (PVI) with bilateral wide area circumferential ablation using radiofrequency ablation. PVI was achieved with lesions placed 5-10 mm outside the veno-atrial junction aiming for isolation as ipsilateral PV pairs. The anterior border of the left PVs was ablated on the LA appendage ridge where possible, or on the appendage side of the ridge for cases where this was unstable. Lesions were delivered on the venous side of the appendage ridge only where this was necessary to isolate PVs. Ablation was performed with 45W, 15 seconds anteriorly and 12 seconds posteriorly. Further ablation was performed if AF organized into an AT or there was previous documentation of AT.

1. ***CV methodology***

A local gradient method was used to calculate CV. To reduce the effects of spatial resolution when comparing results across cases, each electroanatomic mapping mesh was first re-meshed to an average resolution of 2mm using Meshtool software (12), and all calculations were performed on this mesh. The LAT measurements were interpolated to give a LAT for every node on the mesh. This was achieved using an inverse distance weighting interpolation through Meshtool software. The gradient of the interpolated activation times was then used to estimate CV for each element of the mesh through utilizing the xyz coordinates and determining the distance between each element.

1. ***Pivot points***

A novel wavefront tracking algorithm developed and executed in Matlab (Mathworks, MA, USA) was used offline to track wavefront propagation in SR. All patients had 30-seconds unipolar recordings collected sequentially using the HD-grid catheter. To allow effective tracking of the wavefront using the algorithm, the sequential recordings overlapped, ensuring each segment had several overlapping recordings in each direction. Combining multiple overlapping unipolar recordings allowed a prediction of the wavefront propagation over an anatomical segment and over time to be determined through matching wavefront patterns obtained for each unipolar recording.

Raw unipolar electrograms, electrode xyz coordinates and geometry data were utilized. Firstly, ventricular far field signals were filtered. Atrial signals were then annotated. Filtering of far field ventricular and atrial signals were evaluated manually to ensure accurate exclusion of far field signals and atrial signal annotation. Inaccurate atrial annotations due to noise or fractionated signals were excluded from the analysis.

Electrodes were then paired to their neighboring electrodes through comparing geodesic distance between electrodes. The atrial activations were then compared amongst these electrodes to track the wavefront propagation. The fixed atrial pacing enabled establishment of predicted wavefront propagation.
