# Supplementary figures and images for: Autonomic modulation impacts conduction velocity dynamics and wavefront propagation in the left atrium
Source: Europace. 2024 Sep 4;26(9):euae219. doi: 10.1093/europace/euae219 (PMC11372476; doi:10.1093/europace/euae219)

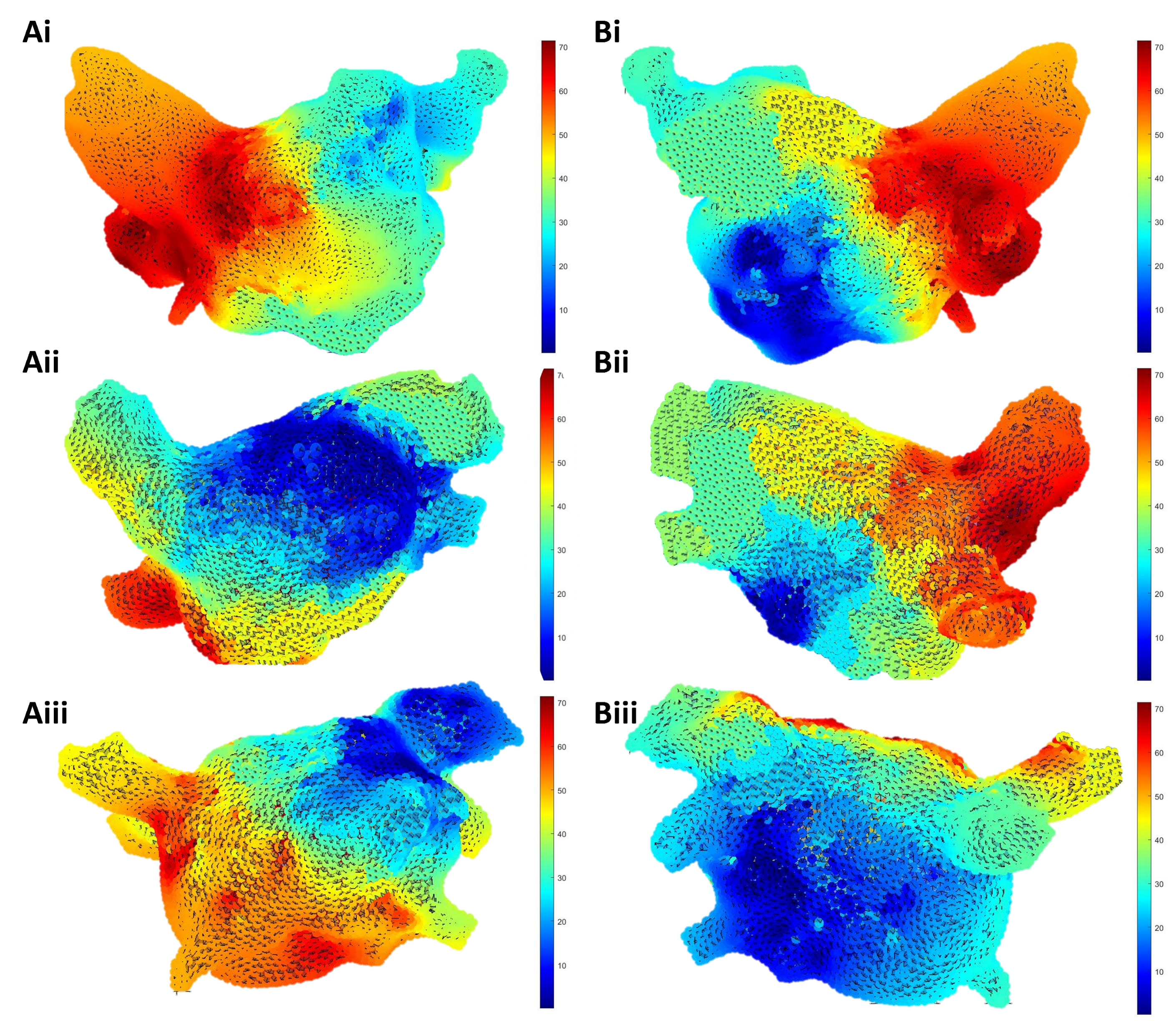

Supplement: euae219_Supplementary_Data [file euae219_supplementary_data.zip › SupplementalFigure1A-Bfinal.tif]
